# Draft Genome Sequence of Probiotic *Enterococcus faecium* Strain L-3

**DOI:** 10.1128/genomeA.01622-15

**Published:** 2016-01-28

**Authors:** Alena Karaseva, Anna Tsapieva, Justin Pachebat, Alexander Suvorov

**Affiliations:** a FSBSI Institute of Experimental Medicine, St. Petersburg, Russian Federation; b Institute of Biological, Environmental and Rural Sciences, Aberystwyth University, Aberystwyth, Ceredigion, United Kingdom; c St. Petersburg State University, St. Petersburg, Russian Federation

## Abstract

We report here the draft genome sequence of the bacteriocin producer *Enterococcus faecium* strain L-3, isolated from a probiotic preparation, Laminolact, which is widely used in the Russian Federation. The draft genome sequence is composed of 74 contigs for a total of 2,643,001 bp, with 2,646 coding genes. Five clusters for bacteriocin production were found.

## GENOME ANNOUNCEMENT

Enterococci are Gram-positive lactic acid bacteria that inhabit the gastrointestinal tracts of diverse hosts (1–3). On one hand, the species *Enterococcus faecium* was considered a harmless commensal of the mammalian gastrointestinal (GI) tract and is commonly used as a probiotic (4) and in food products (5); on another hand, some enterococcal strains were associated with infection, especially in immunocompromised patients (6, 7). *E. faecium* strain L-3 was isolated from a probiotic preparation. This strain has a wide range of antimicrobial activities against organisms, including *Listeria monocytogenes*, *Streptococcus pyogenes*, and *Streptococcus agalactiae* (8).

Genomic DNA was extracted from the cultured bacterium by the DNeasy blood and tissue kit (Qiagen, USA). The genome was sequenced *de novo* using 454 pyrosequencing on the GS FLX platform (Roche 454 Life Sciences). Barcoded DNA libraries were prepared using the GS DNA library preparation kit in combination with GS multiplex identifiers kits. The GS emulsion-based clonal amplification (emPCR) kit II was used for the Multiplex Identifier-adapted library. GS LR70 sequencing kit in combination with GS FLX PicoTiterPlate kit (70 × 75 bp) was used to obtain the sequences. All procedures were carried out according to the manufacturer’s instructions (Roche). Reads were *in silico* assembled using the Newbler assembler, giving 74 contigs of different sizes (smallest contig, 102 bp; largest contig, 211,071 bp). Genome annotation was performed using the standard operation procedures from the Rapid Annotations using Subsystems Technology (RAST) server (9).

The draft genome sequence consists of 2,643,001 bases, with a mean of G+C content of 38%. A total of 2,646 coding sequences (CDSs) and 60 structural RNAs were predicted. There were 331 RAST subsystems determined in the genome, which represents only 50% of the sequences assigned.

Several CDSs for the production of bacteriocins, namely, enterocin A, enterocin B, two-component enterocin X (X-alfa and X-beta subunits), and lactobin-like bacteriocins, were found.

Seventeen contigs (total size of 115,433 bp) were recognized as a part of a plasmid related to the rep17 family (10).

The sequence was analyzed for the presence of genes that are considered to be virulence markers employing PCR. The genes for gelatinase (*gelE*), serine protease (*sprE*), quorum-sensing regulator (*fsrB*), putative adherence factors (*asa1*, *efaA*, and *esp*), cytolysins (*cylA*, *cylB*, *cylM*, and *cylL*), hemolysin (*hyl*), and genes related to vancomycin resistance (*vanA*) were not found in this strain (11). The sequences of these genes have not been determined in any contigs under study.

### Nucleotide sequence accession numbers.

This whole-genome shotgun project has been deposited at DDBJ/EMBL/GenBank under the accession number JRGX00000000. The version described in this paper is version JRGX01000000.
